# Underprescription of Fibrate Among Patients With Diabetic Retinopathy in Perak, Malaysia

**DOI:** 10.7759/cureus.51434

**Published:** 2024-01-01

**Authors:** Jazlan Jamaluddin, Mohd Azzahi Mohamed Kamel

**Affiliations:** 1 Family Medicine, Klinik Kesihatan Selayang Baru, Selangor, MYS; 2 Family Medicine, Klinik Kesihatan Lenggong, Perak, MYS

**Keywords:** fibrate, malaysia, registries, diabetic retinopathy, fibric acids, prescriptions

## Abstract

Objectives

Diabetic retinopathy (DR) is a major cause of blindness and its prevalence is increasing. Fibrate, specifically fenofibrate, has been shown to be efficacious in reducing the progression of DR. This study aims to determine the five-year trend of and factors associated with the prescription of fibrate among patients with DR in Perak.

Methods

Data on all patients with DR in 76 government health clinics in Perak who were audited between 2018 and 2022 were extracted from the National Diabetes Registry (NDR), excluding those who were lost to follow-up. Multivariable logistic regression was used to identify factors associated with the prescription of fibrates.

Results

Data from 4028 patients were analysed. Commonly prescribed medications were statins (n = 3466, 86.0%), metformin (n = 3212, 79.7%), and angiotensin-converting enzyme inhibitors (n = 2318, 57.5%). Only 63 (1.6%) patients were prescribed fibrate. Factors associated with the prescription of fibrates were patients from the clinics in northern (adjusted odds ratio (aOR) = 0.33, 95% CI: 0.12-0.65) and southern clusters (aOR = 0.23, 95% CI: 0.08-0.655), triglycerides > 1.7 mmol/L (aOR = 4.85, 95% CI: 1.85-12.70), and prescription of insulin (aOR = 2.77, 95% CI: 1.07-7.18) and statin (aOR = 0.10, 95% CI: 0.04-0.27).

Conclusion

The prescription of fibrate among patients with DR was low, highlighting a missed opportunity for early treatment and improved outcomes in primary care. The prescription of fibrates to reduce the progression of DR should be expanded to primary care. Clinicians should consider the factors associated with the non-prescription of fibrate identified when prescribing to these patients. Policies, including those at the ministry level, to enhance the availability of these medicines, including financial resources for procurement, are necessary to guarantee easy access for patients in different areas. It is crucial for healthcare providers to be knowledgeable about and follow guidelines. Moreover, improving the overall management of DR in patients with multiple comorbidities can be achieved by addressing worries about the side effects of combination therapies through educational campaigns and providing clear directives. Nevertheless, the study's findings should be interpreted in light of the limitations discussed.

## Introduction

Diabetes mellitus (DM) is a major public health problem that is approaching epidemic proportions in Malaysia. Based on the latest National Health and Morbidity Survey (NHMS) in 2019, the overall prevalence of raised blood glucose among adults aged 18 years and older was 18.3%. More worryingly, the prevalence of known diabetes was only 9.4% [1]. Diabetic retinopathy (DR), a known complication of DM, is one of the leading causes of blindness and visual disability in adults [2]. In Malaysia, the National Diabetes Registry (NDR) Report 2020 has shown an increasing proportion of patients diagnosed with retinopathy within one year, from 10.6% in 2019 to 11.5% in 2020 [3]. A similar trend was also observed in the state of Perak, where the proportion of patients with DR increased from 11.0% in 2019 to 12.7% in 2020. A report from the Diabetes Eye Registry and studies from the Klang Valley region and tertiary care centres in the state of Kelantan have shown that the prevalence of DR among type 2 DM (T2DM) patients is 11.8-39.3% [4-6]. DR prevalence is closely linked to the duration of DM [7]. At the time of diagnosis, fewer than 5% will develop retinopathy, but after 10 years, the proportion increases to 40-50%. After 20 years of T2DM, around 60% of patients with diabetes will develop some degree of retinopathy. As patients are being diagnosed with DM at a younger age, more patients are expected to develop complications such as DR. Treatment of DR usually involves anti-vascular endothelial growth factor (anti-VEGF) injections, laser photocoagulation, and vitrectomy, which are costly and may lead to various adverse effects [8]. As such, prevention is better than cure. These complications can be avoided with early detection, proper follow-up, and timely intervention.

Fibrates, specifically fenofibrate, have been shown to slow the progression of DR in T2DM patients and reduce the need for more invasive treatments such as laser photocoagulation and vitrectomy [9,10]. Essentially, patient acceptance of an oral pharmacotherapy such as fenofibrate would be higher as compared to an invasive procedure such as laser treatment. Evidence for the efficacy of fenofibrate in slowing the progression of DR comes from the results of two large randomized controlled trials (RCTs): Action to Control Cardiovascular Risk in Diabetes (ACCORD) and Fenofibrate Intervention and Event Lowering in Diabetes (FIELD) [11,12]. A meta-analysis of these RCTs has shown that fenofibrate treatment reduced the need for retinal laser treatment by over 20% compared with placebo and this benefit may accumulate over time [13]. The economic burden of DR increases with worsening severity. The healthcare costs of patients who undergo vitrectomy are higher than those who undergo photocoagulation. By maintaining patients in the mild stages of DR for longer, fenofibrate monotherapy or combination with statin was projected to improve quality-adjusted life expectancy versus placebo or statin alone. Direct medical costs were marginally higher for fenofibrate therapy compared to placebo or statins but were partially offset by reduced laser surgery and vitrectomy associated with advanced DR.

The use of fibrates in the treatment of DR is not new. Many international guidelines, such as the diabetes guidelines by the Royal College of Ophthalmologists of the United Kingdom, the American Diabetes Association, and Singapore’s Ministry of Health (MOH) Clinical Practice Guidelines (CPG) on the management of DM recommended the prescription of fenofibrate to halt DR, particularly in patients with significant hyperlipidaemia since 2012, 2013, and 2014, respectively [14-16]. In Malaysia, the 2011 Malaysian CPG for the screening of DR already recommended the use of fenofibrate to reduce the progression of DR [17]. The latest 2020 CPG on the management of T2DM has again emphasized the use of fenofibrate to slow the progression of retinopathy, irrespective of baseline dyslipidaemia status [8]. The use of fenofibrate to prevent the progression of DR has been approved by MOH Malaysia in 2019. Based on the 2019 NHMS report, 68.2% of those with known diabetes sought treatment at public health clinics or "Klinik Kesihatan (KK)" in primary care [1]. Therefore, an estimated 300 thousand to 1.1 million adults in Malaysia with diabetes have DR and are possibly eligible for fenofibrate in these clinics. Therefore, this study aims to look at the prescription of fibrate among patients with DR. Factors associated with the prescription of fibrates identified could help clinicians and policymakers to identify and widen access to this medication for this high-risk population.

This study was previously presented as an oral presentation at the 25th Family Medicine Scientific Conference, Malaysia on August 27, 2023.

## Materials and methods

This was a cross-sectional study using data from the NDR for five years in Perak, which is one of the 13 states in Malaysia. All adults aged 18 years old and above with DR selected for audit in NDR from 2018 to 2022 were included. Those who were lost to follow-up or for whom data were not available when selected for audit were excluded. The data were extracted from NDR, a surveillance web-based registry database used to track diabetes management and clinical outcomes in patients receiving care from KKs in Malaysia. There are registry and clinical audit datasets in the database [3]. The registry dataset is regularly updated with new diabetes patients recorded and when patients are lost to follow-up or passed away. Every year, a selection of patients is randomly selected for clinical audits. Annual clinical audits were undertaken in August, with T2DM patients from all KKs randomly chosen. Regardless of whether they had previously been audited, all active patients had an equal chance of being sampled. In Perak, data for NDR were collected from 76 participating KKs from all 11 districts. The clinical management of patients in KKs is usually monitored by family medicine specialists (FMS), either in-house or remotely. For this study, data for sociodemographics, clinical characteristics, and medication prescriptions were extracted and analysed. Since medications were categorized in the NDR by their medication class, data for fenofibrate prescriptions are included in the fibrate class. Therefore, the outcome, which was non-prescription of fibrate, was defined as fibrate not being prescribed among patients with DR during the NDR audit of that year. For demographic characteristics, patients were divided into clusters according to the KKs that they were followed up in. The cluster corresponds to the cluster hospital concept that started in 2017. It involves the combination of at least one specialist hospital with the nearest non-specialist hospitals and KKs in the same geographical area, forming a single entity with shared resources. The Ipoh cluster in Perak involves hospitals and KKs in Kinta and Kampar districts. The southern cluster includes Batang Padang, Hilir Perak, Manjung, Muallim, and Perak Tengah districts. The northern cluster involves Kuala Kangsar, Hulu Perak, Larut, Matang dan Selama, and Kerian districts. Other patients’ clinical characteristics were categorized according to the recommendations of local guidelines [8,18].

The sample size was calculated using OpenEpi, version 3.01, update 47 on 06/04/2013, for "Sample Size for Frequency in a Population". Approximately 4000 patients with DR were audited in a year in NDR and the percentage of patients with a prescription of fibrate is 3.8% based on a cohort study by Meer et al. [19]. Therefore, to estimate the prevalence of fibrate use among patients with DR with an absolute precision of ±5% and a two-sided 95% confidence interval, only 57 patients are required to be studied. However, all adult patients with DR selected for audit in NDR from 2018 to 2022 based on inclusion and exclusion criteria were included in this study.

Data of patients with DR in the NDR were de-identified and analysed as a cohort using IBM SPSS Statistics for Windows, version 27.0. (IBM Corp., Armonk, NY). Data were summarized using numbers and percentages. Normality testing was determined by the Shapiro-Wilk test. Since all numerical data were found to be non-normally distributed, median with interquartile ranges (IQR) were used. Missing data were treated with pairwise deletion in subsequent analyses. Difference testing between groups was performed using the chi-square test or Fisher’s exact test, as appropriate. To determine independent factors associated with the prescription of fibrate among patients with DR, a multivariable logistic regression analysis was performed with the prescription of fibrate as the dependent variable. To reduce the number of variables included in the multivariable model, a simultaneous entry method approach was used with an inclusion criterion of P-value <0.25. Collinearity between variables was ruled out before covariates were introduced in the model. The goodness of fit was tested using a Hosmer and Lemeshow test, and odds ratios (OR) with 95% confidence intervals (CIs) were computed. All reported P-values are two-sided and a P-value < 0.05 is considered statistically significant.

## Results

**Figure 1:**
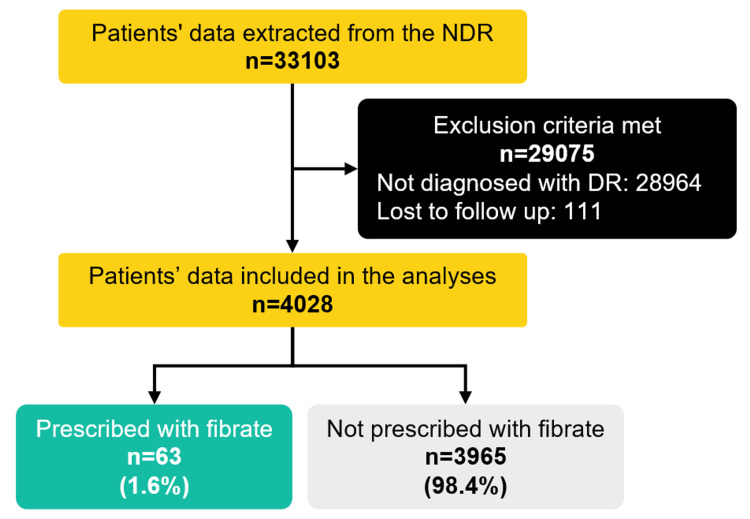
Flow diagram of the study. NDR: National Diabetes Registry; DR: diabetic retinopathy.

A total of 4139 or 13.1% (95% CI: 12.8-13.5) of audited patients from NDR were diagnosed with DR. From these, only 4028 patients fulfilled the inclusion and exclusion criteria (Figure 1).

Most of the patients with DR are of female gender (N = 2529, 62.8%), Malay ethnicity (N = 2735, 67.9%), and diagnosed with hypertension (N = 3623, 90.2%) and dyslipidaemia (N = 3574, 89.3%). The median age of the patients was 65.0 years with 13.0 years of diabetes and 4.0 years of retinopathy (Table 1).

**Table 1:** Sociodemographic characteristics of patients in general and according to the prescription of fibrate (n = 4028). * Fisher's exact test.

Sociodemographic characteristics	n (%) or median (IQR)	Prescription of fibrate, n (%)		P-value
		No	Yes	
Cluster				<0.001
Ipoh	678 (16.8)	653 (16.5)	25 (39.7)	
Southern	1530 (38.0)	1519 (38.3)	11 (17.5)	
Northern	1820 (45.2)	1793 (45.2)	27 (42.9)	
Age, years	65.0 (13.0)			0.044
<60	1199 (29.8)	1173 (29.6)	26 (41.3)	
≥60	2829 (70.2)	2792 (70.4)	37 (58.7)	
Sex				0.144
Female	2529 (62.8)	2495 (62.9)	34 (54.0)	
Male	1499 (37.2)	1470 (37.1)	29 (46.0)	
Ethnicity				0.081
Chinese	647 (16.1)	640 (16.1)	7 (11.1)	
Malay	2735 (67.9)	2693 (67.9)	42 (66.4)	
Indian	601 (14.9)	588 (14.8)	13 (20.6)	
Orang Asli (Peninsular)	9 (0.2)	8 (0.2)	1 (1.6)	
Others	36 (0.9)	36 (0.9)	0 (0)	
Duration of diabetes, years	11.1 (7.9)			0.724
<10	1686 (41.9)	1661 (41.9)	25 (39.7)	
≥10	2342 (58.1)	2304 (58.1)	38 (60.3)	
Age of diabetes diagnosis, year	54.0 (15.0)			0.022
<60	2864 (71.1)	2811 (70.9)	53 (84.1)	
≥60	1164 (28.9)	1154 (29.1)	10 (15.9)	
Duration of retinopathy, years	4.0 (6.6)			0.004
<5	2284 (56.7)	2237 (56.4)	47 (74.6)	
≥5	1744 (43.3)	1728 (43.6)	16 (25.4)	
Comorbidities				
Hypertension	3623 (90.3)	3563 (90.2)	60 (95.2)	0.179
Dyslipidaemia	3574 (89.3)	3512 (89.2)	62 (98.4)	0.018
Ischemic heart disease	368 (9.5)	362 (9.5)	6 (9.5)	0.994
Cerebrovascular disease	152 (3.9)	149 (3.9)	3 (4.8)	0.737*
Nephropathy	1193 (30.6)	1165 (30.3)	28 (44.4)	0.016
Diabetic foot ulcer	145 (3.8)	143 (3.8)	2 (3.2)	1.000*
Amputation	114 (3.0)	112 (2.9)	2 (3.2)	0.710*
Erectile dysfunction	156 (25.6)	154 (25.6)	2 (28.6)	1.000*
Smoker	160 (3.9)	150 (6.1)	5 (11.4)	0.191*

The majority of them were obese (N = 1422, 41.0%) with elevated waist circumference (N = 1828, 74.2%). Most of them have a blood pressure of <140/80. The median glycosylated haemoglobin (HbA1c) was 7.7%. Only 1653 (47.5%) of them had low-density lipoprotein cholesterol (LDL-C) < 2.6. Most of them had normal estimated glomerular filtration rate (eGFR) with no albuminuria (Table 2).

**Table 2:** Clinical characteristics of patients in general and according to the prescription of fibrate. BP: blood pressure; HbA1c: glycosylated haemoglobin; HDL-C: high-density lipoprotein cholesterol; LDL-C: low-density lipoprotein cholesterol; eGFR: estimated glomerular filtration rate.

Clinical characteristics	n (%) or median (IQR)	Prescription of fibrate, n (%)		P-value
		No	Yes	
Waist circumference, cm (n = 2465)	92.0 (15.0)			0.172
Normal	637 (25.8)	622 (25.7)	15 (34.9)	
Elevated (M > 90, F > 80)	1828 (74.2)	1800 (74.3)	28 (65.1)	
Body mass index, kg/m^2 ^(n = 3467)	26.4 (5.0)			0.553
Underweight (<18.5)	623 (18.0)	612 (17.9)	11 (20.4)	
Normal (18.5-22.9)	63 (1.8)	62 (1.8)	1 (1.9)	
Overweight (23.0-27.4)	1359 (39.2)	1334 (39.1)	25 (46.3)	
Obese (≥27.5)	1422 (41.0)	1405 (41.2)	17 (31.5)	
Systolic BP, mmHg (n = 3856)	137.0 (22.0)			0.757
<140	2189 (56.8)	2155 (56.8)	34 (54.8)	
≥140	1667 (43.2)	1639 (43.2)	28 (45.2)	
Diastolic BP, mmHg (n = 3856)	75.0 (14.0)			0.579
≤80	2483 (64.4)	2441 (64.3)	42 (67.7)	
≥80	1373 (35.6)	1353 (35.7)	20 (32.3)	
Random blood glucose, mmol/L (n = 2402)	8.2 (4.2)			0.262
≤8.5	1317 (54.8)	1294 (55.0)	23 (46.9)	
>8.5	1085 (45.2)	1059 (45.0)	26 (53.1)	
Fasting blood glucose, mmol/L (n = 2854)	7.1 (3.5)			0.380
≤7.0	1392 (48.8)	1368 (48.7)	24 (53.3)	
>7.0	1462 (51.2)	1441 (51.3)	21 (46.7)	
HbA1c, % (n = 3839)	7.7 (3.2)			0.502
<7.0	1397 (36.4)	1397 (36.4)	20 (32.3)	
≥7.0	2442 (63.6)	2442 (63.6)	42 (67.7)	
Total cholesterol, mmol/L (n = 3790)	4.6 (1.6)			<0.001
≤5.2	2619 (69.1)	2593 (69.5)	26 (42.6)	
>5.2	1171 (30.9)	1136 (30.5)	35 (57.4)	
Triglycerides, mmol/L (n = 3706)	1.5 (0.9)			<0.001
≤1.7	2393 (64.6)	2371 (65.0)	22 (36.7)	
>1.7	1313 (35.4)	1275 (35.0)	38 (63.3)	
HDL-C, mmol/L (n = 3490)	1.2 (0.5)			0.300
Normal	2201 (63.1)	2167 (63.2)	34 (56.7)	
Low (M < 1.0 and F < 1.3)	1289 (36.9)	1263 (36.8)	26 (43.3)	
LDL-C, mmol/L (n = 3477)	2.6 (1.4)			<0.001
<2.6	1653 (47.5)	1640 (48.0)	13 (22.0)	
≥2.6	1824 (52.5)	1778 (52.0)	46 (78.0)	
Creatinine, mmol/L (n = 3747)	84.5 (40.0)			
eGFR categories, ml/min/1.73m² (n = 3747)	75.4 (39.2)			0.932
G1 (≥90)	1179 (31.5)	1162 (31.5)	17 (28.3)	
G2 (60-89)	1402 (37.4)	1379 (37.4)	23 (38.3)	
G3a (45-59)	599 (16.0)	589 (16.0)	10 (16.7)	
G3b (30-44)	343 (9.2)	338 (9.2)	5 (8.3)	
G4 (15-29)	161 (4.3)	158 (4.3)	3 (5.0)	
G5 (<15)	63 (1.7)	61 (1.7)	2 (3.3)	
Urine protein (n = 3036)				0.090
Negative	1849 (60.9)	1826 (61.1)	23 (48.9)	
Positive	1187 (39.1)	1163 (38.9)	24 (51.1)	
Urine microalbumin (n = 2287)				0.422
Negative	1469 (64.2)	1450 (64.3)	19 (57.6)	
Positive	818 (35.8)	804 (35.7)	14 (42.4)	

**Figure 2:**
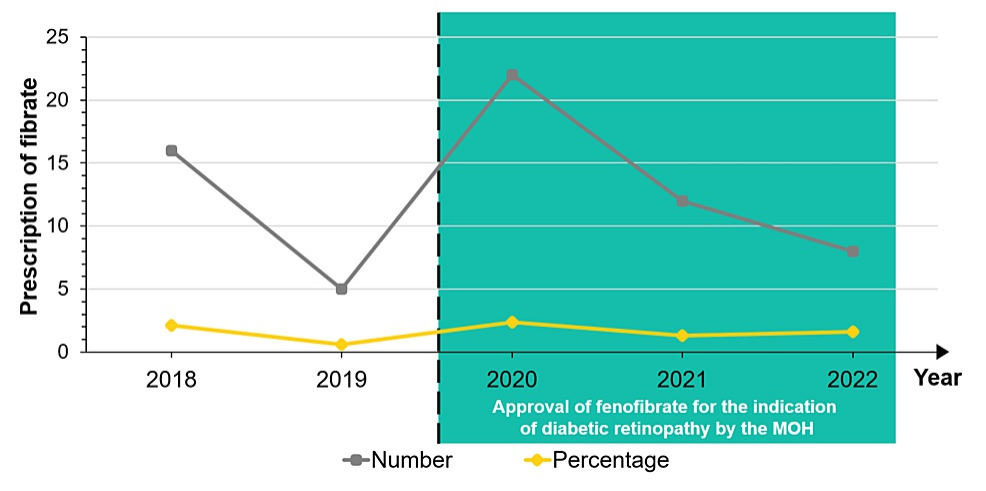
Trend of fibrate prescription from 2018 to 2022. MOH: Ministry of Health.

Patients with DR were prescribed mostly metformin (N = 3212, 79.7%) and sulphonylureas (N = 1751, 43.5%) as glucose-lowering drugs, angiotensin-converting enzyme inhibitors (N = 2318, 57.5%) and calcium channel blockers (N = 2396, 56.9%) as antihypertensive, and statin (N = 3466, 86.0%) as lipid-lowering drugs. Only 63 (1.6%) patients with DR were prescribed fibrates during this five-year period (Table 3). The trend of prescription has remained relatively the same. There was no statistical difference in the cumulative number of fibrate prescriptions for DR after approval of its use in the MOH (P = 0.206) (Figure 2).

**Table 3:** Medications prescribed for patients with diabetes retinopathy and according to the prescription of fibrate (n = 4028). * Fisher's exact test. ACE: angiotensin-converting enzyme; ARB: angiotensin II receptor blockers.

Medications	n (%)	Prescription of fibrate, n (%)		P-value
		No	Yes	
Glucose-lowering drugs (GLDs)				
Metformin	3212 (79.7)	3159 (79.7)	53 (84.1)	0.383
Sulphonylureas	1751 (43.5)	1727 (43.6)	24 (38.1)	0.753
Alpha-glucosidase inhibitors	78 (1.9)	77 (1.9)	1 (1.6)	1.000*
Meglitinides	4 (0.1)	4 (0.1)	0 (0)	1.000*
Glitazones	47 (1.2)	47 (1.2)	0 (0)	1.000*
Insulin	1682 (41.8)	1647 (41.5)	35 (55.6)	0.025
Anti-platelets				
Acetyl salicylate acid	1301 (32.3)	1281 (32.3)	20 (31.7)	0.009
Ticlopidine	31 (0.8)	31 (0.8)	0 (0)	1.000*
Anti-hypertensives				
ACE inhibitors	2318 (57.5)	2279 (57.5)	39 (61.9)	0.497
ARB	483 (12.0)	478 (12.1)	5 (7.9)	0.997
Calcium channel blockers	2396 (59.5)	2355 (59.4)	41 (65.1)	0.362
Diuretics	1050 (26.1)	1027 (25.9)	23 (36.5)	0.057
Beta-blockers	1252 (31.1)	1227 (30.9)	25 (39.7)	0.137
Alpha-blockers	205 (5.1)	201 (5.1)	4 (6.3)	0.647
Centrally acting	15 (0.4)	15 (0.4)	0 (0)	1.000
Lipid-lowering drugs				
Statins	3466 (86.0)	3429 (86.5)	37 (58.7)	<0.001
Fibrates	63 (1.6)	-	-	-

Univariate logistic regression analysis was performed, and the following variables were found to have a P-value < 0.25: age, sex, ethnicity, cluster, age of diabetes diagnosis, duration of retinopathy, smoking status, diagnosis of nephropathy, hypertension, dyslipidaemia, waist circumference, total cholesterol, triglyceride, LDL-C, urine protein, insulin, beta-blockers, diuretics, and statins. From the multivariable logistic regression analysis, factors associated with non-prescription of fibrates were patients from the clinics in northern (adjusted odds ratio (aOR) = 0.23, 95% CI: 0.12-0.87) and southern clusters (aOR = 0.23, 95% CI: 0.08-0.65), triglycerides > 1.7 (aOR = 4.85, 95% CI: 1.85-12.70), and prescription of insulin (aOR = 2.77, 95% CI: 1.07-7.18) and statin (aOR = 0.10, 95% CI: 0.04-0.27) (Table 4).

**Table 4:** Factors associated with the prescription of fibrate among patients with diabetic retinopathy. The model reasonably fits well (Hosmer−Lemeshow test: chi-square = 11.48;P = 0.176); model assumptions were met; no significant interactions and multicollinearity problem; model explained between 5.3% (Cox and Snell R^2^) and 27.8% (Nagelkerke R^2^) of the variance for non-prescription of fibrate. The model correctly classified 87.4% of cases with a sensitivity of 6.9% and a specificity of 99.8%. * Statistical significance at P < 0.05. aOR: adjusted odds ratio; CI: confidence interval.

Variables	Adjusted beta	Wald statistics (df)	aOR (95% CI)	P-value
Cluster				
Ipoh			Reference	
Southern	-1.547	7.570 (1)	0.23 (0.08-0.65)	0.006*
Northern	-1.135	5.030 (1)	0.33 (0.12-0.87)	0.025*
Triglycerides, mmol/L				
≤1.7			Reference	
>1.7	1.605	5.233 (1)	4.85 (1.85–12.70)	0.001*
Insulin				
No			Reference	
Yes	1.005	4.403 (1)	2.77 (1.07-7.18)	0.036*
Statins				
No			Reference	
Yes	-2.283	21.476 (1)	0.10 (0.04-0.27)	<0.001*

## Discussion

This study found that fibrate, including fenofibrate, was not being prescribed adequately to patients with DR in public healthcare settings in Malaysia, and this underprescription has remained unchanged since the medication was approved for DR indication. A previous study done in Malaysia has shown that the prevalence of non-proliferative DR, where fenofibrate is most effective, among patients with DR is approximately 87.3% [20-22]. Considering some of these patients may be contraindicated for fibrates, such as those with chronic kidney disease, gallstone disease, and pancreatitis, an estimated 80% of DR patients are expected to require a fenofibrate prescription. Nevertheless, this study aligns with earlier studies conducted in the United States and Taiwan, which reported low prescription rates of fenofibrates for DR at 3.9% and 7.8%, respectively [19,23]. Most of the research conducted on the prescription of fibrates to patients with DR relies on cohort or hospital data, with a lack of studies in primary care for direct comparison. The limited accessibility and availability of fibrates in primary care facilities could be one factor contributing to the underprescription. There is also a potential lack of awareness of the benefits of fibrates for patients with DR among clinicians due to the limited availability and accessibility of fibrates in KKs. Despite the fact that numerous CPGs have recommended using fenofibrate in patients with established retinopathy since 2011, its prescription in the MOH facilities for the indication of DR, specifically for the reduction of DR progression, is only allowed to be initiated by endocrinologists and ophthalmologists (A* category) [8,17]. However, early DR, including mild to moderate non-proliferative DR without maculopathy, can be followed up in primary care and these patients should ideally be initiated with fenofibrate early for the best outcome [8,11-13,17]. The current restriction on the prescriber’s category has and will continuously delay the initiation of fenofibrate, leading to worse overall outcomes for DR and potential blindness. Medications for prevention and reduction of complications progression should be approved for initiation in primary care during medication approval submission itself and started early in primary care as primary care plays a monumental role in preventive and holistic care. Therefore, the prescription for this medication should not be limited to endocrinologists and ophthalmologists only.

Fenofibrate also needs to be started for the long term to sustain the benefit in patients with DR. A follow-on study of the ACCORD cohort found that the protective effect of fenofibrate did not persist after the cessation of fenofibrate, unlike the metabolic memory effect observed with prior intensive glycaemic control [24]. Therefore, the cost of fenofibrate can be a significant barrier to its prescription. In the United States (US), a study has shown that although fenofibrate accounts for only 65.2% of fibrate use, it accounts for a disproportionate 78.7% of the cost [25]. Since medications in KKs are provided effectively complimentary for its citizens, the cost of medications can limit the ability for these clinics to purchase them, which in turn may reduce the prescription of medications even if patients are indicated for them. Due to the limited financial resources, KKs may prioritize other essential expenses over the cost of medications, leading to the underprescription of fibrates. A systematic review has shown that the cost of the medications was mostly mentioned to influence physicians’ prescribing decisions after the characteristics of the medications [26]. Other studies have also shown that the cost of medications can be a significant factor in medication adherence to prescription [27,28]. Therefore, a cheaper price for fibrate would possibly increase its prescription among patients with DR and provide potential long-term benefits. Furthermore, policymakers can explore ways to reduce the cost of medications for patients, such as through subsidies or other financial assistance programs, as treatment of fenofibrate has been found to be cost-effective in reducing retinopathy progression [29].

This study found that patients with DR from the clinics in the northern and southern clusters in Perak were more likely not to be prescribed fibrates. Since fibrates must be prescribed by an FMS in KKs, delays in the initiation of fibrates, especially in rural areas with less accessible specialist care, including FMS, are expected. Studies have also shown that patients managed by specialists showed better risk factor control and better follow-up [30,31]. The restriction on fibrate prescriptions for the indication of DR to endocrinologists or ophthalmologists, which are even less accessible, would certainly worsen this situation. The lack of awareness or knowledge of the guidelines and recommendations for prescribing fibrates in patients with DR could also contribute to underprescription. Furthermore, patient-related factors such as poor adherence to medication, contraindications, or intolerance to lipid-lowering drugs could also contribute to underprescription [32].

This study has shown that patients with triglycerides > 1.7 are more likely for fibrate prescription. Fibrates are primarily prescribed to patients with high triglyceride levels, as it has been shown to reduce triglycerides by 20-35%, compared to statins at 6-30% [8,18]. Therefore, it is not surprising that patients with DR who have higher triglyceride levels are more likely to be prescribed fibrates compared to those with normal triglyceride levels. Since evidence for fibrate in the reduction of cardiovascular disease (CVD) events is limited and conflicting, it is recommended in the guidelines mainly for the treatment of patients with very high triglyceride levels who do not respond to non-pharmacological measures rather than for its cardiovascular effect [8,18]. However, considering the additional benefits of fenofibrate in patients with DR, its prescription in KKs should not be limited to the hypertriglyceridemia indication only and it should be the first-line treatment for patients with early DR.

The study also found that patients who were prescribed insulin were more likely to be prescribed fibrates. Patients who are prescribed insulin are likely to have multiple comorbidities such as hypertension, nephropathy, and CVD, which are also risk factors for DR [17]. Therefore, clinicians may be more aware of the need to prescribe fibrates in these patients to prevent or slow down the progression of DR. Moreover, patients who are prescribed insulin may have worse diabetes control or a more advanced DR [17]. In these cases, clinicians may be more likely to prescribe fibrates as a part of a comprehensive treatment plan. Furthermore, patients who are prescribed insulin may have more frequent follow-up visits with clinicians [8]. This provides an opportunity for clinicians to assess the patient's overall health and risk factors and prescribe fibrates as needed.

Lastly, the prescription of statin was found to be 90% less likely to be prescribed fibrates. Although these patients were indicated for a fibrate prescription, clinicians may be concerned about potential side effects since most patients will be on two different lipid-lowering agents. As shown in this study, 86.0% of patients with DR were already on statins. Adding fibrates to these patients may potentially increase the side effects of medications. Fibrates have previously been shown to increase the risk of myopathy with statins, and the risk is highest for gemfibrozil. Thus, the combination of statins and gemfibrozil is discouraged by local guidelines [18]. Nevertheless, meta-analyses have shown no significant difference in muscle-related adverse events between treatment with statins or fibrates alone and in combination with fibrates [18]. This is especially true for the combination of statins with fenofibrate as the risk seems to be small [18]. Therefore, it is important to consider individual patient factors in the decision-making process for prescribing fibrates, including fenofibrate in combination with statins. It also underscores the need for clinicians to be aware of the benefits of fibrates in managing DR and cardiovascular risk factors.

This study carries several important implications for clinical practice and healthcare policy. Firstly, the consistently low prescription rates of fibrates among DR patients, especially in certain geographic clusters, highlight potential gaps in accessibility and awareness. The limited availability of fenofibrates in primary care settings, as suggested by this study, may contribute to underprescription. Addressing this issue could involve improving the distribution of these medications or finances to ensure that they are readily accessible to healthcare providers in various regions. Secondly, this study underscores the importance of healthcare provider awareness and adherence to CPG. The fact that certain patients, such as those prescribed insulin and statins, were more likely to receive fibrates suggests that there might be a nuanced understanding of the risk factors and comorbidities that warrant fibrate prescription. Encouraging consistent adherence to guidelines, especially among primary care physicians, can contribute to more uniform and evidence-based care. Furthermore, the study implies a potential gap in understanding the interaction between different medications. The lower likelihood of prescribing fibrates in patients already on statins may reflect concerns about potential adverse effects or drug interactions. Addressing these concerns through educational initiatives and clear guidelines on combination therapies could enhance the overall management of DR in patients with multiple comorbidities. The study also calls attention to the need for a more comprehensive and nuanced approach to DR management. The focus on fibrates, without differentiation between advanced and early DR or an assessment of the effectiveness of fibrates in managing DR, leaves important questions unanswered. Future research could explore these aspects to guide a more tailored and effective approach to DR treatment. These implications should inform future strategies aimed at optimizing the care of individuals at risk of or affected by DR.

The main strength of the study is the use of registry-based data from the NDR, which is a comprehensive database of diabetic patients registered in public healthcare facilities in Malaysia. This allowed for a large sample size and provided a representative sample of patients with DR in Perak. This study used a retrospective cross-sectional design, which allowed for the real-life examination of clinicians' management of patients with DR over a five-year period, enabling the investigation of trends in fibrate prescriptions over time. Another strength of the study is the focus on fibrates prescription, which is a vital medication for the management of DR. The study's findings highlight the underprescription of fibrates in Perak, which could have significant implications for the management of DR and the prevention of vision loss in diabetic patients. Furthermore, the study's findings are consistent with previous research on the underprescription of medications for DR. This provides support for the study's conclusions and reinforces the importance of addressing the barriers to fibrates' prescription to improve the management of DR and reduce the risk of vision loss among diabetic patients.

This study has several limitations. The data are extracted from the NDR, which only includes patients registered with public healthcare facilities, potentially limiting the generalizability of the findings. Although most patients with diabetes were followed up in KK based on the 2019 NHMS report, excluding those from hospital and private healthcare facilities could potentially limit the generalizability of the study's findings. Although audited patients were randomly selected, selection bias may still exist. The accuracy of data is also dependent on the healthcare provider who entered it, which may lead to incomplete or inaccurate information and limit the validity of the findings. However, regular audits of the datasets are being done continuously to verify and address any potential weaknesses when using the registry. The study only analysed fibrates and not specifically fenofibrate due to the limitation of the registry, potentially limiting the findings since fenofibrate is the only fibrate proven to reduce the progression of DR. However, a local study has shown that fenofibrate is the most commonly prescribed fibrate in primary care, similar to studies in the US and Canada [25,33]. This study did not analyse other reasons for the underprescription of fibrates or differentiate between advanced and early DR in its analysis, which could impact the effectiveness of fibrates. Additionally, the study did not investigate the effectiveness of fibrates in managing DR, which would be useful in determining the potential benefits of increasing prescription. Future studies addressing these limitations would provide a more comprehensive understanding of the issue.

## Conclusions

The prescription of fibrate among patients with DR in this study was low, highlighting a missed opportunity for early treatment and improved outcomes in primary care. The prescription of fibrates to reduce the progression of DR should be expanded to primary care, especially in KKs. Clinicians should consider the factors associated with the non-prescription of fibrate identified when prescribing to these patients. Policies, including those at the ministry level, to enhance the availability of these medicines, including financial resources for procurement, are necessary to guarantee easy access for patients in different areas. It is crucial for healthcare providers to be knowledgeable about and follow CPG. Moreover, improving the overall management of DR in patients with multiple comorbidities can be achieved by addressing worries about the side effects of combination therapies through educational campaigns and providing clear directives. Nevertheless, the study's findings should be interpreted in light of the limitations discussed.
